# The Defective and Dependent

**Published:** 1911-12-02

**Authors:** 


### THE DEFECTIVE AND DEPENDENT.

### III. Mentally Defective Children.

### ASYLUMS FOR IDIOTS AND IMBECILES.

The movement inaugurated in England in 1847 was followed by the formation of other charitable institutions for idiots and imbeciles in various parts of the kingdom. We have already noted the development, from the original foundation at Highgate, of the two large asylums at Earlswood and Colchester, the former receiving patients from all parts of the British Dominions, the latter from the Eastern Counties of England. In 1864 the Western Counties Asylum for Idiots was instituted at Starcross, Devon, and in 1868 the Midland Counties Asylum at Knowle, near Birmingham. In 1870 the Royal Albert Asylum at Lancaster (now the largest voluntary institution in England) began to receive patients from the northern counties of England; and in 1873 the first Poor Law Training Institution for Imbecile Children was established by the Metropolitan Asylums Board, subsequently developing into the large asylum at Darenth. In Scotland two charitable institutions for imbecile children were formed at Larbert (near Stirling) and Baldovan (near Dundee) respectively; and in Ireland the Stewart Institution, near Dublin, came into operation. In the provinces in a few instances special wards have been provided for idiotic and imbecile children in connection with county lunatic asylums. Thus in one way and another accommodation now exists in England for special treatment in residential institutions of some 3,000 children and young persons of the imbecile class, recognised by the Lunacy Commissioners. Although these institutions were originally known as "Asylums for Idiots and Imbeciles," it must be remembered that for many years they were the only agencies for dealing with all grades of mental defect, and that at least 20 per cent. of their inmates were of the class nowadays designated "feeble-minded," a term indeed denoting incapacity only differing in degree from that of the imbecile. The admission to such institutions has, however, been regulated by the so-called Idiots Act of 1886, which requires all inmates to be certified as "idiots or imbeciles from birth or from an early age."

### Schools for Mentally Defective Children.

With the passing of the Education Act of 1870 and the universally compulsory school attendance which it entailed, it soon became evident that a considerable number of pupils, scarcely to be described as imbecile, were in certain respects not of normal mental constitution and were consequently incapable of benefiting by the curriculum of the ordinary elementary school. A series of investigations carried on by Dr. Francis Warner and a committee of experts, under the auspices of the Charity Organisation Society, the British Medical Association, the British Association for the Advancement of Science, and other scientific bodies, between the years 1888 and 1894, estimated, from an examination of 100,000 school children in and about London, that the proportion requiring exceptional modes of education by reason of physical or mental defect approximated to 1 per cent. of the aggregate. The Royal Commission on the Blind, Deaf and Dumb, etc., which reported in 1889, had recommended with regard to "feeble-minded children" that they "should be separated from the ordinary scholars in public elementary schools in order that they may receive special instruction, and that the attention of school authorities be particularly directed towards this object." In London and several provincial towns special classes for children deemed defective were subsequently established, the first being actually opened at Leicester in April 1892, and others in London in the same year, the system being that each teacher should have a comparatively small number of children to deal with, and that manual and physical training should form a leading part of the curriculum. These classes necessarily entailed a considerable additional charge, and, the Education Authorities who had taken up the matter pressing for an additional Government grant, the Education Department (now Board of Education) appointed a Committee to consider the special arrangements desirable in the case of defective and epileptic children, and in consequence of their report the Education Act of 1899, providing for that class was passed.

In this Act "defective children" were defined as "children who not being imbecile and not being merely dull and backward, are defective"—that is to say, "children who by reason of mental or physical defect are incapable of receiving proper benefit from the instruction in an ordinary public elementary school, but are not incapable, by reason of such defect, of receiving benefit from instruction in a certified special class or school." A certificate to this effect is to be given in each case by a qualified medical practitioner approved by the Board of Education, in order to secure the additional grant (the amount of which is £4 to £4 10s. per annum), and compulsory attendance at a special class or school is required in such cases up to 16 years of age. Unfortunately the Act is merely permissive, and up to the present time has been adopted by only 142 out of the 322 school authorities in England and Wales. On July 31, 1911, there were 169 certified schools for the mentally defective, with an aggregate of 12,253 children on their registers, the majority of the schools being in London and other large towns. In country districts with scattered populations it is, of course, impracticable to form special classes available for day scholars, and although, with the consent of the parents, arrangements may be made for mentally defective children to be boarded out in convenient proximity to special schools of other authorities, or to be sent to residential schools for special instruction, it seems highly desirable that compulsory powers for this purpose should be granted, as is already the case with regard to epileptics.

